# Characterization of the S100A1 Protein Binding Site on TRPC6 C-Terminus

**DOI:** 10.1371/journal.pone.0062677

**Published:** 2013-05-03

**Authors:** Jan Bily, Lenka Grycova, Blanka Holendova, Michaela Jirku, Hana Janouskova, Kristyna Bousova, Jan Teisinger

**Affiliations:** Department of Protein Structures, Institute of Physiology, Academy of Sciences of the Czech Republic, Prague, Czech Republic; Centro Nacional de Biotecnologia - CSIC, Spain

## Abstract

The transient receptor potential (TRP) protein superfamily consists of seven major groups, among them the “canonical TRP” family. The TRPC proteins are calcium-permeable nonselective cation channels activated after the emptying of intracellular calcium stores and appear to be gated by various types of messengers. The TRPC6 channel has been shown to be expressed in various tissues and cells, where it modulates the calcium level in response to external signals. Calcium binding proteins such as Calmodulin or the family of S100A proteins are regulators of TRPC channels. Here we characterized the overlapping integrative binding site for S100A1 at the C-tail of TRPC6, which is also able to accomodate various ligands such as Calmodulin and phosphatidyl-inositol-(4,5)-bisphosphate. Several positively charged amino acid residues (Arg852, Lys856, Lys859, Arg860 and Arg864) were determined by fluorescence anisotropy measurements for their participation in the calcium-dependent binding of S100A1 to the C terminus of TRPC6. The triple mutation Arg852/Lys859/Arg860 exhibited significant disruption of the binding of S100A1 to TRPC6. This indicates a unique involvement of these three basic residues in the integrative overlapping binding site for S100A1 on the C tail of TRPC6.

## Introduction

TRPC6 is a member of a large group of transient receptor potential (TRP) channels, a diverse group of cation-permeable channels. This family includes more than 30 proteins that play critical roles in various biological processes [1]. They are widely expressed in the nervous system and non-excitable cells. TRP channels are involved in many physiological processes, they are important regulators of cation homeostasis in cells and many of them serve as biological sensors for the detection of various environmental stimuli [2]. TRP channels can be divided into seven subfamilies (C - canonical, V - vanilloid, M - melastatin, ML - mucolipin, P - polycystin, A – ankyrin, N – no mechanoreceptor potential C) on the basis of sequence homology [3]. TRP channels contain six putative transmembrane domains, a pore region between domain 5 and 6, and amino- and carboxy- intracellular termini [3], [4]. Many conserved domains have been identified within the intracellular termini, and they serve as important interaction sites for various regulatory molecules [2].

Members of the TRPC subfamily (TRPC1-TRPC7) typically contain 3–4 ankyrin repeats on their N-terminus and a conserved region called TRP box at the C-terminus, also present in TRPVs and TRPMs. These channels are non-selectively permeable to cations, nevertheless their selectivity for calcium over sodium varies among their members. It has been proposed that TRPC channels can be activated by the stimulation of phospholipase C, but a number of different modulators such as signaling proteins, cytoskeletal elements, scaffold molecules and also other ion channels have been reported to modulate their activity. All TRPC channels have been described as store-operated channels (SOCs), that are activated when intracellular Ca^2+^ stores become depleted [5].

TRPC6 was reported to be modulated by Ca^2+^ ions together with calmodulin (CaM) [6], [7], [8]. The CaM binding region on the C-terminus was described in detail [6], [8]. The domain contains several consensus CaM binding motifs with hydrophobic residues in specific positions and was also shown to interact with the IP3 receptor (IP3R) and described as the so-called CaM and IP3R binding domain (CIRB) conserved among all TRPC members [6].

S100A proteins are a large family of calcium-binding proteins found in *Vertebrates*. It was assumed that these proteins simply function as cellular calcium buffers, but they have been later reported to take part in the regulation of many cellular processes [9].

S100A1 is a small dimeric protein that is highly expressed in cardiomyocytes, and also in fast and slow twitch muscles, brain and hippocampal neurons [10]. It contains two EF-hand motifs that are able to bind Ca^2+^ ions, causing a conformational change by moving one helix and exposing a broad hydrophobic surface that enables the protein to interact with a variety of target proteins and ion channels [11], [12]. In contrast to CaM, the first EF hand motif binds Ca^2+^ with a lower affinity than the second EF hand [13], [14].

The TRPC6 channel modulates the calcium levels in eukaryotic cells, including sensory receptor cells in response to external signals. Its activity is modulated by multiple factors. It was recently shown that CaM, a Ca^2+^ binding protein, acts as an important mediator of Ca^2+^-dependent regulation [6], [8] and the interaction site was characterized [7], [8]. S100A1 behaves similarly in terms of mediating the activity of other ion channels [15], [16]. For this reason we tested the binding of the S100A1 protein to TRPC6 C-terminus (CT) (801–878), which interacts directly with CaM [7], [8]. Using site-directed mutagenesis of the predicted basic amino acid residues of TPRC6 CT combined with steady-state fluorescence anisotropy measurements, we found that the S100A1 protein is able to bind to the TRPC6 C-terminus. Moreover, this binding site overlaps with the previously reported CIRB binding site on the same receptor.

## Materials and Methods

### TRPC6 Cloning, Expression and Purification

The coding region for the C-terminal part of rat TRPC6 (amino acids 801–878) was cloned into the pET42b expression vector (Novagen). Point mutations of several basic amino acids, namely Arg852, Arg864, Lys 856 and Ile 857, as well as the triple substitutions Arg852/Lys859/Arg860 and Lys859/Arg860/Arg864 were performed by site-directed mutagenesis according to the manufacturer’s protocol (Stratagene). All the constructs were confirmed by DNA sequencing.

The proteins were expressed as fusion proteins with a His-tag on their C-termini in *E. coli* Rosetta cells (Novagen). The expression of the proteins was induced by 0.5 mM isopropyl-1-thio-β-D-galactopyranoside for 16 hours at 20°C. The proteins were purified by Chelating Sepharose Fast Flow (Amersham Biosciences) according to the manufacturer’s protocol. Gel permeation chromatography on Superdex 75 (Amersham Biosciences) was used as a final purification step. A 25 mM Tris-HCl buffer (pH 7.5) containing 500 mM NaCl and 2 mM CaCl_2_ was used for the elution. The protein samples were concentrated using spin columns (Millipore) and the purity was verified by 15% SDS-PAGE (Fig. S1). The concentration of the constructs was estimated by measuring absorbance at 280 nm. The integrity of the proteins was verified by MS-MS analysis. The expression and purification of the (His)6-tagged proteins was described in detail previously [17].

### S100A1 Protein Cloning, Expression, Purification and Labeling

cDNA coding for human S100A1 was cloned into the pET28b expression vector (Novagen). The protein was expressed in E.coli BL21 cells. Its expression was induced by 0.5 mM isopropyl-1-thio-b-D-galactopyranoside for 12 hours at 25°C. The protein was purified using Phenyl Sepharose CL4B (Amersham Biosciences) equilibrated with 50 mM Tris-HCl (pH 7.5), 100 mM NaCl, 2 mM CaCl_2_. Bound S100A1 was eluted with 50 mM Tris-HCl (pH 7.5), 100 mM NaCl, 1.5 mM EDTA and concentrated using a spin column for protein concentration (Millipore). Concentrated protein solution was loaded into a Superdex 75 column (Amersham Pharmacia Biotech) and eluted with 25 mM Tris-HCl (pH 7.5), 100 mM NaCl, 2 mM CaCl_2_ as a final purification step. The purity of the protein was verified by 15% SDS-PAGE (Fig. S2). The concentration of the protein was assessed by BCA’s assay using BSA as a standard protein [18].

The S100A1 protein was then dialyzed against 10 mM NaHCO_3_ buffer (pH 10.0) and 100 mM NaCl for 8 hours at 4°C. The protein sample was mixed with 0.6 dansyl chloride (DNS) solution (Sigma) in a molar ratio of 1∶1.5 and incubated at 4°C for 12 h. The sample was dialyzed for 8 hours against 25 mM Tris-HCl (pH 7.5), 150 mM NaCl and 2 mM EDTA to remove the free DNS-chloride and then against the same buffer containing 2 mM CaCl_2_ instead of EDTA at 4°C overnight [19]. The incorporation stoichiometry was determined by comparing the peak protein absorbance at 280 nm with the absorbance of the bound DNS measured at 340 nm, using an extinction coefficient of 4,300 m^−1^ cm^−1^T [20].

### Steady-state Fluorescence Anisotropy Measurements

Steady-state fluorescence anisotropy measurements were used as a binding assay to determine the binding affinity between fluorescently labeled S100A1 protein and the C-terminal part of TRPC6 and its mutants. The experiment was performed using an ISS PC1™ photon-counting spectrofluorimeter The monochromator excitation and emission wavelengths were set to 340 nm and 520 nm for all measurements. The fraction of bound TRPC6 protein (FB) was calculated from the equation 1:
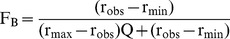
where r_obs_ is the observed anisotropy for any TRPC6 protein concentration, r_max_ is the anisotropy at saturation and r_min_ is the minimum observed anisotropy for the free DNS-S100A1. The parameter Q represents the quantum yield ratio of the bound to the free form, and was estimated by calculating the ratio of the intensities of the bound to the free fluorophore. To determine the equilibrium dissociation constant (K_D_), the values of the bound fraction of the protein were plotted against the TRPC6 protein concentration and fitted using the equation 2:



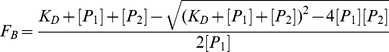
where K_D_ is the equilibrium dissociation constant, [P1] is the DNS-S100A1 concentration, and [P2] is the concentration of the TRPC6 protein. Non-linear data fitting was performed using the program SigmaPlot10 [17]. All experiments were carried out in at least triplicate.

### Mass Spectrometric Analysis

The excised protein band from SDS-PAGE, TRPC6, was digested with trypsin endoprotease (Promega) directly in the gel after destaining and cysteine modification by iodoacetamide [21]. The resulting peptide mixture was extracted, loaded onto the MALDI-TOF target with α-cyano-4-hydroxycinnamic acid as the matrix, and positively charged spectra or MS/MS were acquired using an UltraFLEX III mass spectrometer (Bruker-Daltonics, Bremen, Germany) with internal calibration (monoisotopic [M+H]^+^ ions of the TRPC6 peptides with known sequences).

### Circular Dichroism Spectroscopy

Circular dichroism (CD) experiments were carried out in a Jasco J-815 spectrometer (Tokyo, Japan). The protein concentration was kept constant for all measured samples and was 0.35 mg/ml. The spectra were collected from 200 to 300 nm using a 0.1 cm quartz cell at room temperature. A 0.5 nm step resolution, 20 nm/min speed, 8 s response time and 1 nm bandwidth were used. After baseline correction, the final spectra were expressed as a molar elipticity Q (deg·cm^2^·dmol^–1^) per residue. Secondary structure content was estimated using Dichroweb software [22].

## Results and Discussion

### TRPC6 and S100A1 Expression and Purification

**Figure 1:**
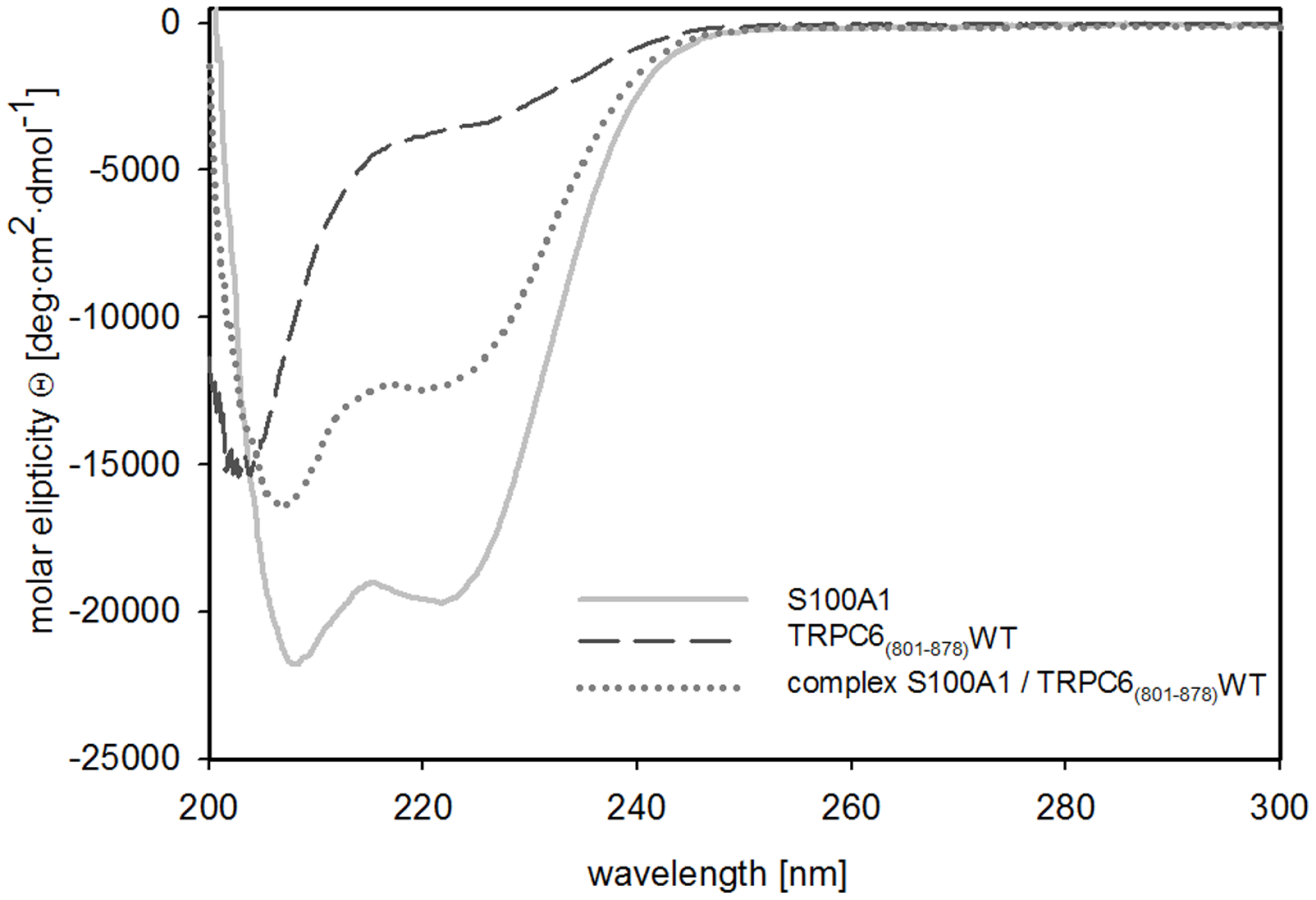
Circular dichroism spectroscopy measurement of TRPC6_(801–878)_WT, S100A1 and the complex of S100A1 and TRPC6 _(801–878)_WT. Examples of CD spectra of TRPC6_(801–878)_WT, S100A1 and the complex of S100A1 and TRPC6 _(801–878)_WT expressed as a molar elipticity Q (deg·cm^2^·dmol^–1^) per residue.

The TRPC6 C-terminal protein construct (801–878) and its mutants were expressed as fusion proteins with a 6x His-tag on their C-termini in E. coli Rosetta cells. The S100A1 protein was expressed in E.coli BL21 cells. All proteins were purified by a two-step purification process. The proteins were soluble and in sufficient amount to perform the binding experiments (Fig. S1 and S2). The integrity of the proteins was verified by circular dichroism spectroscopy measurement. Numerical analysis of the experimental spectra enabled estimation of the relative abundance of the various secondary structure elements. (Fig. 1, Tab. 1). The α-helical conformation (66%) was found to be the major component of the S100A1 protein, which is in good agreement with the conformation found in its native state. The structure of the TRPC6 C-terminus is unknown. According to the theoretical prediction of the secondary structural elements using computational tools, the region was predicted to be mostly unordered. The CD spectra analysis confirmed the theoretical prediction, suggesting that the TRPC6 protein construct was adopting its native form. The experiment was also used to observe changes in the secondary structural elements during the creation of the TRPC6/S100A1 complex (Fig 1, Tab 1). We compared the CD spectrum of the complex with the CD spectra of the proteins alone. Because the CD spectra of the mixture are the sum of the TRPC6 protein construct and S100A1 individually, we suggest that the changes in the secondary structure of TRPC6 (801–878) have no significant effect on its binding to S100A1.

**Table 1:** Calculated incidence (%) of secondary structures of S100A1 and TRPC6_(801–878)_ WT and TRPC6 single mutant R852A and complex of TRPC6_(801–878)_ WT/S100A1.

Protein	Helix	Antiparallel	Parallel	Beta Turn	Random Coil
TRPC6_(801–878)_WT	0,18	0,15	0,12	0,20	0,36
TRPC6_(801–878)_ R852A	0,22	0,14	0,11	0,20	0,34
S100A1	0,66	0,03	0,03	0,12	0,15
S100A1+ TRPC6_(801–878)_WT	0,43	0,07	0,07	0,17	0,25

### S100A1 Binds to TRPC6 (801–878) C-terminus

S100 protein family is known to act as calcium-signaling molecules by converting changes in cellular calcium levels to a variety of biological responses. In this manner, many of the S100 proteins have been shown to modulate enzyme activities, oligomerization of cytoskeletal protein components (tubulin, desmin, glial fibrillary acidic protein), modulate ubiquitination, control membrane vesicle formation and participate in trafficking of proteins to the inner surface of the plasma membrane [11]. The interaction between the transient receptor potential cation channel proteins TRPV5 and TRPV6 with S100A10 has been shown using two-hybrid and co-immunoprecipitation experiments [23], [24]. In this role, the S100A10-annexin A2 complex is thought mediate trafficking of the TRV5 and TRV6 proteins to the plasma membrane where they act as calcium-selective channels. Recently our research group has also shown a direct interaction between S100A1 protein and TRPM3 ion channel, where this protein was able to compete with CaM for the overlapping binding site [25].

**Figure 2:**
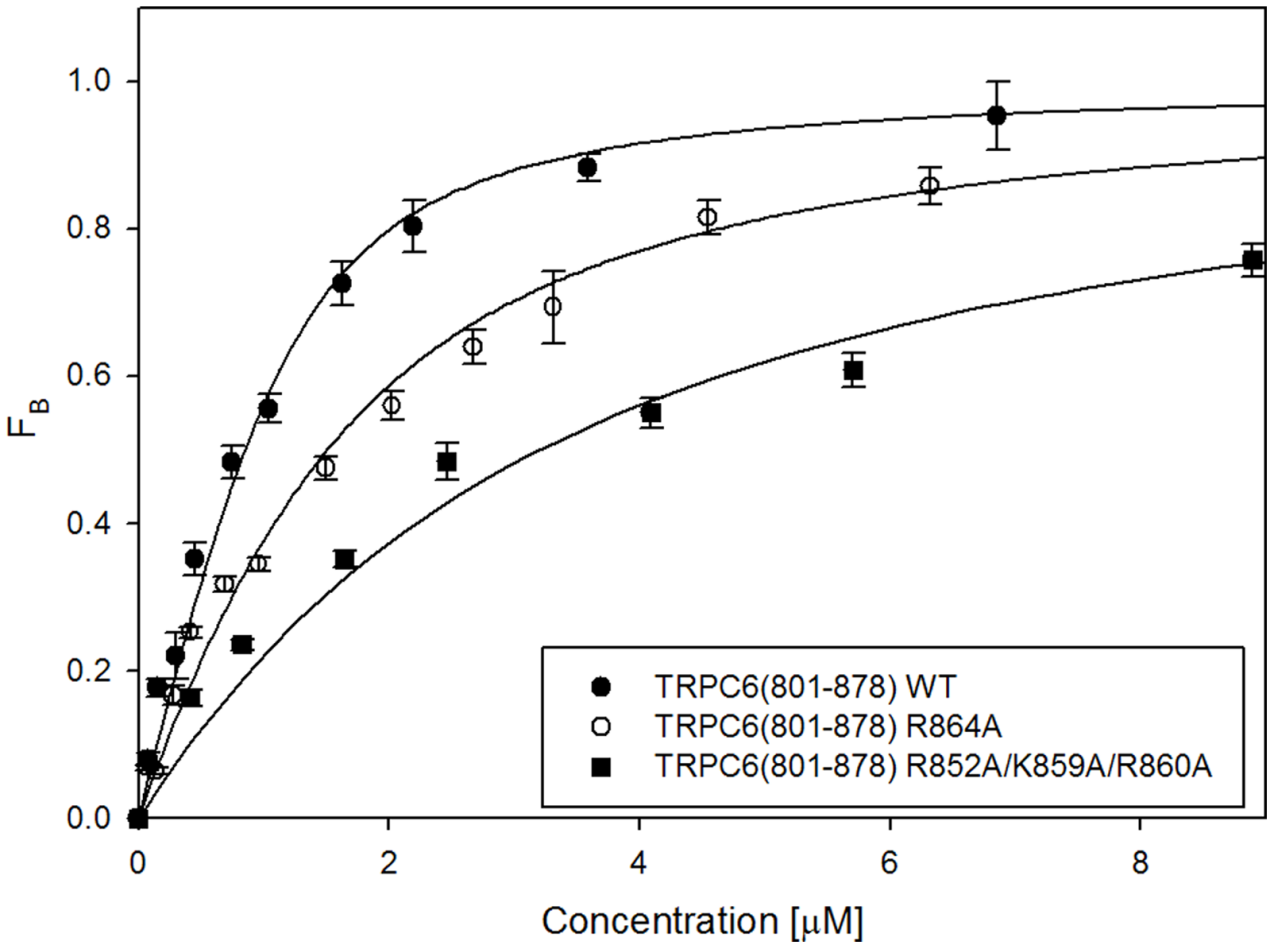
Steady-state fluorescence anisotropy measurement of TRPC6_(801–878)_ WT and selected mutants to fluorescently labeled S100A1 protein. DNS- S100A1 protein (232 µM) was titrated with TRPC6 fusion protein and the Fb was calculated using equation 1 as was described in material and methods.Binding isotherms and dissociatin constants were calculated by fitting the data to the equation 2 as was described in material and methods. Values are expressed as the mean ± standard deviation (SD) measured from at least from three independent experiments. Binding isotherms of wild-type TRPC6 _(801–878)_ is represented as black circles, single mutant is TRPC6_(801–878)_ R864A as white circles and triple mutant TRPC6_(801–878)_ K859A/R860A/R864A as black squares.

**Figure 3:**
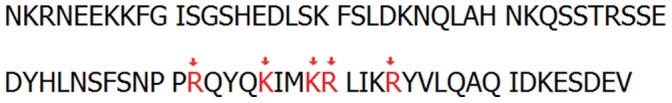
Amino acid senquence of TRPC6 fusion protein. Native rat TRPC6 801–878 amino acid sequence containing integrative binding site was investigated. Predicted important basic amino acids that were replaced by alanine are in red.

The protein samples were used for steady-state fluorescence anisotropy measurement to characterize the binding ability of the S100A1 protein to TRPC6 C-terminal region 801–878. Increasing amounts of the TRPC6 protein construct were titrated into the DNS-S100A1 solution. The equilibrium dissociation constant of the TRPC6_(801–878)_/Ca^2+^-S100A1 complex was estimated to be 0.31+/−0.04 µM (Fig. 2). The value of the equilibrium dissociation constant is nearly the same as was estimated for CaM binding to be 0.320+/−0.019 µM [8]. As the amino acid residues important for CaM binding are known (Fig. 3), we tested their role in S100A1 binding (Fig. 2). The single substitution of R852A and triple substitution of K859A/R860A/R864A had almost no effect on its binding. (Fig. 2, Tab. 2). Interestingly, in comparison to the CaM binding, where the neutralization of this residue had the most striking effect [8], the R852 residue did not influence the interaction with S100A1 at all. Although the TRPC6 WT binding affinities to both ligands CaM and S100A1 are almost the same, according to the results we obtained it seems that different residues are involved in the binding. The mutations of K856A and R864A lead to an up to 3-fold decrease in the binding affinity to S100A1. These results are in a good agreement with TRPC6/CaM binding data. The R852A/K859A/R860A triple mutation caused the most significant decrease in binding ability (Tab. 2).

**Table 2:** Summary of estimated equilibrium dissociation constants of the complex of TRPC6_(801–878)_WT and its mutants with S100A1.

Protein	K_D/µ_M
TRPC6_(801–878)_ *WT*	0.31+−0.04
TRPC6_(801–878)_ R852A	0.48+−0.11
TRPC6_(801–878)_ K856A	0.76+−0.09
TRPC6_(801–878)_ R864A	0.92+−0.08
TRPC6_(801–878)_R852A/K859A/R860A	2.62+−0.22
TRPC6_(801–878)_K859A/R860A/R864A	0.40+−0.05

### Binding of TRPC6 801–878 to S100A1 is Calcium-dependent

**Figure 4:**
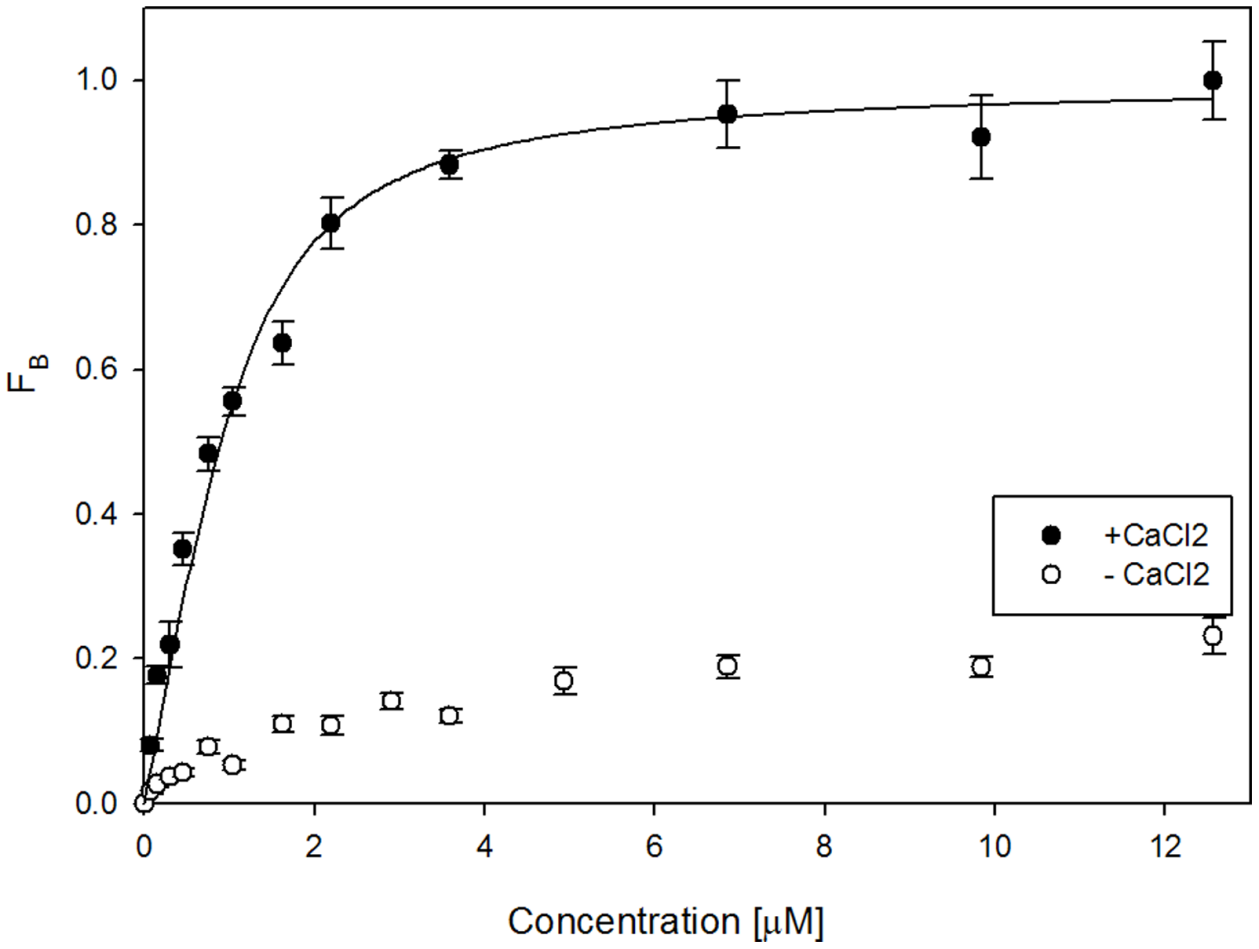
Steady-state fluorescence anisotropy measurement of interaction between TRPC6_(801–878)_ WT and DNS- S100A1 protein in presence and absence of calcium ions. Titration of DNS-S100A1 protein (232 µM) with TRPC6 fusion protein in presence of calcium ions resulted in an increase of bound fraction (Fb) (white dots) compared to when the DNS-S100A1 protein was titrated with TRPC6 fusion protein in absence of of calcium ions (black dots). Values are expressed as the mean ± standard deviation (SD) measured from at least three independent experiments.

Since S100A1 is a Ca^2+^-binding protein and Ca^2+^ ions play a crucial role in TRPC6 activity regulation, the role of calcium ions in S100A1 binding to TRPC6 (801–878) was assessed. The experiment was performed in a solution without calcium. There was no increase in fluorescence anisotropy when calcium was absent (Fig. 4), suggesting that the binding is calcium-dependent. The same behavior has been detected for the CaM/TRPC6– CT (801–878) interaction [8].

In this report the interaction of the Ca^2+^-binding protein S100A1 with the conserved region (801–878) of the TRPC6-CT was investigated. We found that these proteins bind with high affinity and the binding is Ca^2+^-dependent, analogous to that of CaM. According to our results, the interaction is similar but not exactly the same, because different residues are essential for the interaction. Here we show the role of some basic amino acid residues from the so-called CIRB region of TRPC6 affecting its binding to S100A1. These results could suggest potential physiological consequences which will need further investigation.

## Supporting Information

Figure S2 **Final purification step of the S100A1 protein.** Chromatogram and SDS-PAGE of fractions 1-6 after gel chromatography on Sephadex 75.
(DOCX)

Figure S1 **Final purification step of the protein construct TRPC6 (801–878).** Chromatogram and SDS-PAGE of fractions 1–9 after the gel chromatography on Sephadex 75.
(DOCX)
