# Supplementary figures and images for: Characterization of the S100A1 Protein Binding Site on TRPC6 C-Terminus
Source: PLoS One. 2013 May 3;8(5):e62677. doi: 10.1371/journal.pone.0062677 (PMC3643951; doi:10.1371/journal.pone.0062677)

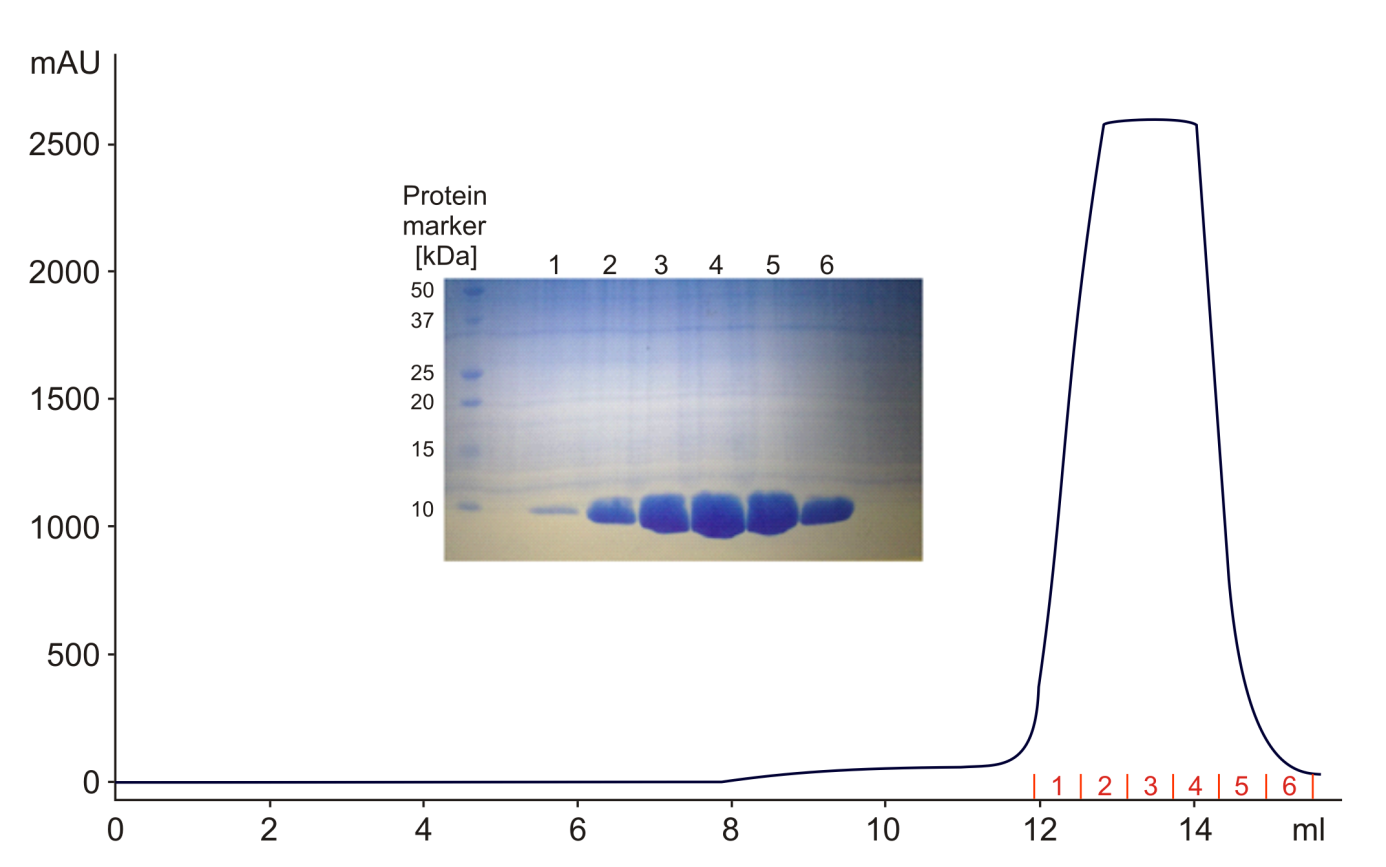

Supplement: Figure S2 — Final purification step of the S100A1 protein. Chromatogram and SDS-PAGE of fractions 1-6 after gel chromatography on Sephadex 75. (DOCX) [file pone.0062677.s001.docx]

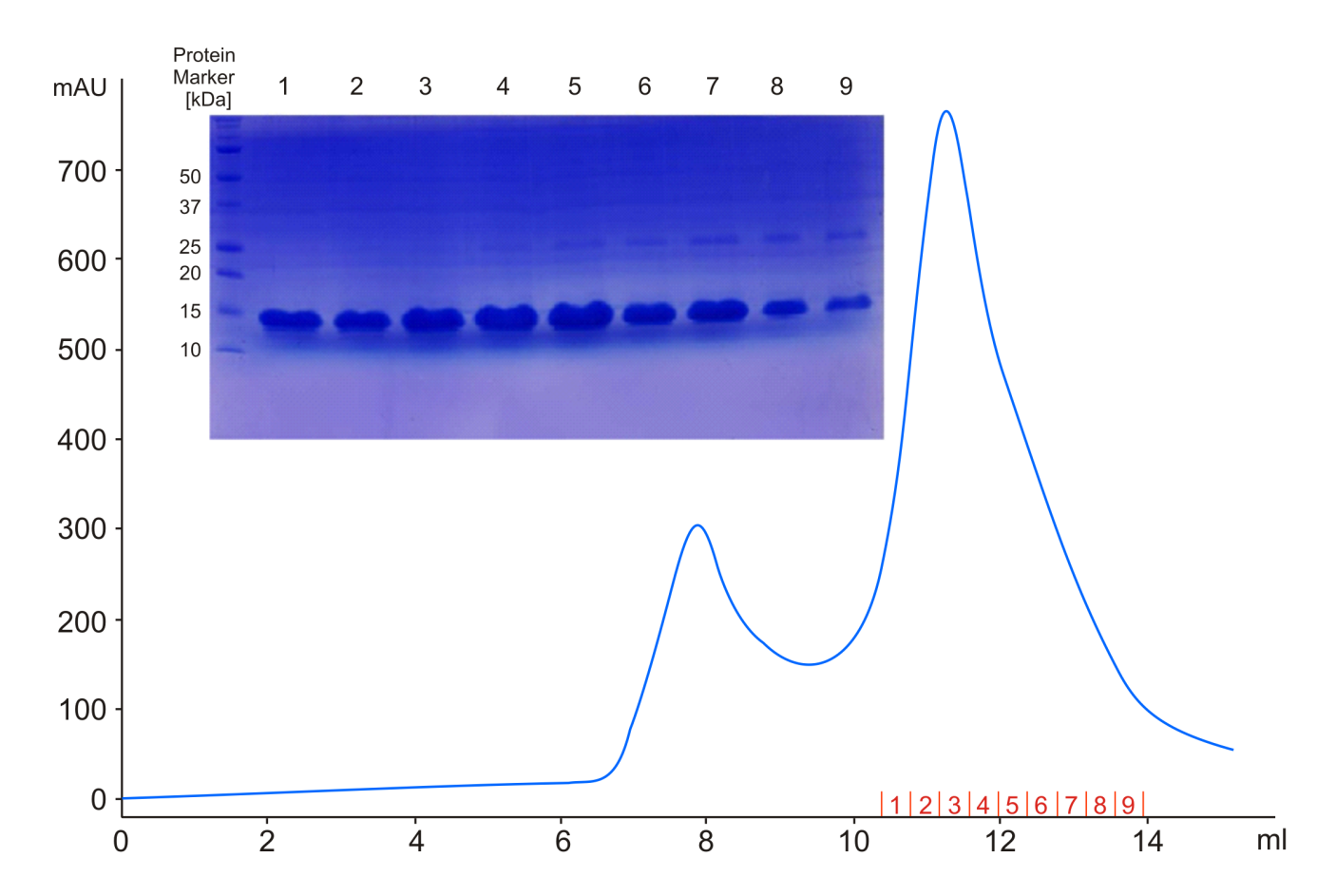

Supplement: Figure S1 — Final purification step of the protein construct TRPC6 (801–878). Chromatogram and SDS-PAGE of fractions 1–9 after the gel chromatography on Sephadex 75. (DOCX) [file pone.0062677.s002.docx]
